# MiR-886-3p Regulates Cell Proliferation and Migration, and Is Dysregulated in Familial Non-Medullary Thyroid Cancer

**DOI:** 10.1371/journal.pone.0024717

**Published:** 2011-10-05

**Authors:** Yin Xiong, Lisa Zhang, Alisha K. Holloway, Xiaolin Wu, Ling Su, Electron Kebebew

**Affiliations:** 1 Endocrine Oncology Section, Surgery Branch, Center for Cancer Research, National Cancer Institute, Bethesda, Maryland, United States of America; 2 Gladstone Institutes, University of California San Francisco, San Francisco, California, United States of America; 3 Laboratory of Molecular Technology, Science Applications International Coorporation-Frederick, Inc., National Cancer Institute, Frederick, Maryland, United States of America; Cardiff University, United Kingdom

## Abstract

**Background:**

The molecular basis and characteristics of familial non-medullary thyroid cancer are poorly understood. In this study, we performed microRNA (miRNA) profiling of familial and sporadic papillary thyroid cancer tumor samples.

**Methodology/Principal Findings:**

Genome wide miRNA profiling of sporadic and familial papillary thyroid cancer was performed. Differentially expressed miRNAs were validated by quantitative RT-PCR. Ectopic expression of miR-886-3p in thyroid cancer lines was performed to identify pathways targeted by the miRNA, as well as, to determine its effect on tumor cell biology. We found four differentially expressed miRNAs between familial and sporadic papillary thyroid cancer tumor samples. MiR-886-3p and miR-20a were validated to be differentially expressed by 3- and 4-fold, respectively. Pathway analysis of genome-wide expression data on cells overexpressing miR-886-3p and target prediction analysis showed genes involved in DNA replication and focal adhesion pathways were regulated by miR-886-3p. Overexpression of miR-886-3p in thyroid cancer cell lines significantly inhibited cellular proliferation, the number and size of spheroids and cellular migration. Additionally, overexpression of miR-886-3p increased the number of cells in S phase.

**Conclusions/Significance:**

Our findings for the first time suggest that miR-886-3p plays an important role in thyroid cancer tumor cell biology and regulates genes involved in DNA replication and focal adhesion. Thus, miR-886-3p may play a role in the initiation and or progression of papillary thyroid cancer.

## Introduction

Thyroid cancer is one of the fastest growing cancer diagnoses in the United States with more than 44,000 new cases estimated to occur in 2011 [1]. Familial non-medullary thyroid cancer (FNMTC) may occur as a minor component of familial cancer syndromes (Gardner's, Cowden's disease, Carney complex type 1, Werner syndrome, McCune-Albright syndrome) or as the predominate feature [2]. Most cases of FNMTC are papillary thyroid cancer and have an autosomal dominant pattern of inheritance with incomplete penetrance. FNMTC accounts for up to 8% of all thyroid cancer cases [2], [3], [4], [5], [6]. In the familial cancer syndromes mentioned above, patients present with distinct extrathyroidal lesions and the susceptibility genes responsible for these syndromes are known. However, the majority (>95%) of FNMTC cases occur as isolated familial nonmedullary thyroid cancer cases for which the susceptibility gene(s) is unknown.

FNMTC is defined as when two or more first-degree relatives are affected with non-medullary thyroid cancer. The genetic basis of FNMTC is poorly understood. In linkage studies, several groups have identified chromosomal loci associated with FNMTC: 1q21, 2q21, 6q22, 8p23.1-p22, and 19p13.2 [7],[8], [9],[10], [11], [12],[13]. Mutation analysis has shown that kindreds with FNMTC do not have germ line mutations in *BRAF*, *RAS*, and *RET/PTC* genes that are commonly mutated somatically in thyroid cancers of follicular cell origin [2]. Furthermore, analysis of somatic mutations (*BRAF*, *RAS*, and *RET/PTC*) in sporadic and familial papillary thyroid cancer tumor samples showed no difference in the rate and type of mutations in these histologies [2], [6], [14]. Thus, it is unknown if the molecular profile of sporadic versus familial thyroid cancer is different. Moreover, it is also unknown if there is a molecular basis for the earlier age of onset and more aggressive disease behavior observed in FNMTC compared to sporadic disease [6], [15], [16].

MicroRNAs (miRNAs) are small (∼21-nucleotide-long) noncoding RNAs, which regulate gene expression and play a significant role in many biological processes, including tumorigenesis [1]–[2]. A specific miRNA may function either as an oncogene or as a tumor suppressor by regulating the expression of target oncogene(s) and tumor suppressor gene(s), respectively. In most cases, miRNAs bind to 3′-untranslated regions (3′-UTRs) of target mRNAs, leading to mRNA degradation or repression of translation. In thyroid cancer, a common single nucleotide polymorphism in pre-miR-146a has been reported to inhibit mature miRNA expression and increase the risk of developing papillary thyroid cancer [17]. To our knowledge, no studies have been performed comparing the miRNA profiles of familial and sporadic cancers in general and specifically for thyroid cancer.

In this study, we performed miRNA profiling of familial and sporadic papillary thyroid cancer tumor samples. We found that miR-886-3p was downregulated in papillary thyroid cancer as compared to normal and differentially expressed in familial papillary thyroid cancer as compared to sporadic papillary thyroid cancer. Pathway analysis of genome-wide expression data on cells overexpressing miR-886-3p and target prediction analysis showed genes involved in the DNA replication and focal adhesion pathway were regulated by miR-886-3p. Indeed, overexpression of miR-886-3p in thyroid cancer cell lines significantly inhibited cellular proliferation, the number and size of spheroids and migration.

## Materials and Methods

### Thyroid tissue samples

Thyroid tissue samples were snap frozen in liquid nitrogen at the time of thyroidectomy. The National Cancer Institute review board approved this research protocol after informed written consent was obtained from all participants. All tissue samples underwent additional histological review by an endocrine pathologist to confirm the diagnosis and identify samples with greater than 80% tumor cells. We used 28 conventional papillary thyroid cancer tumor samples (21 sporadic, 7 familial) and 10 normal thyroid tissue samples for the miRNA array profiling. A family history questionnaire was used to ascertain if tumors should be categorized as sporadic or familial. FNMTC was defined when 2 or more first degree relatives were affected with thyroid cancer of follicular cell origin. Cases of thyroid cancer were confirmed in affected family members. All of the FNMTC tumor samples were from different families. In 5 of 7 tumor samples of FNMTC there were three first degree relatives affected and in 2 of 7 there were two first degree relatives affected. The tumor samples were matched for age (+/−2 years), gender and TNM stage of cancer (at a 3∶1 ratio of sporadic-to-familial cases).

### MiRNA microarray

A total of 28 microarrays (Exiqon™) were run comparing both familial and sporadic papillary thyroid cancers to a pool of normal thyroid tissue. The log_2_ ratio of the Cy5 to Cy3 intensity signals was calculated for each miRNA on every array (with no background subtraction) and the data was normalized by print tip loess normalization [18], [19]. Since individual miRNAs were represented by four probes on the array, the median normalized log_2_ ratio of the replicate probes (for those with more than one unflagged probe) was used as the value for the miRNA. The summarized log_2_ ratios for each experiment were then used in moderated t-statistics and p-value calculation using the limma package in R/Bioconductor [20], [21] with adjustment for false discovery rate using the Benjamini-Hochberg method [22]. MiRNA microarray data, which is MIAME compliant, has been deposited in the GEO database (http://www.ncbi.nlm.nih.gov/geo).

### Cell lines and culture conditions

Human thyroid cancer cell lines FTC-133 (follicular thyroid cancer) and TPC-1 (papillary thyroid cancer) were maintained in Dulbecco's Modified Eagle Medium (DMEM) with 4,500 mg/L D-glucose, L-glutamine, and 110 mg/L sodium pyruvate) supplemented with 10% serum, thyroid-stimulating hormone (TSH) (10 mU/mL), penicillin (10,000 U/mL), streptomycin (10,000 U/mL), Fungizone (250 mg/mL) and insulin (10 (µg/mL) in a standard humidified incubator at 37°C in a 5% CO_2_ and 95% O_2_ atmosphere. Both cell lines are established and authenticated thyroid cancer cell lines [23], [24], [25], [26]. The TPC-1 cell line was provided by Dr. Nabuo Satoh (Japan) and FTC-133 cell line was provided by Dr. Peter Goretzki (Germany). Serum free media (DMEM-F12 media) supplemented with 4 hormones (insulin [10 µg/mL], somatostatin [10 ng/mL], transferrin [5 µg/mL], and hydrocortisone [0.36 ng/mL]) was used for the functional studies.

### MiRNA Transfection

Mature miRNA precursor (pre-miR-886-3p, Applied Biosystems, Foster City, CA) was transfected into cells using Lipofectamine RNAiMAX (Invitrogen, Carlsbad, CA) following the manufacturer's instructions. A random sequence pre-miR (pre-miR-Negative Control) (Applied Biosystems) was used as negative control (miR-NC).

### RNA Isolation and Quantitative real-time RT-PCR

Total RNA was extracted by using TRIzol reagent (Invitrogen) according to the manufacturer's instructions. TaqMan MiRNA Assays (Applied Biosystems) were used to measure miRNA expression level. Total RNA was reverse transcribed with a miRNA-specific primer, followed by real-time PCR with TaqMan probes. U6 was used as the endogenous control. The relative amount of mRNAs was determined using TaqMan Assay (Applied Biosystems) on an ABI 7900 HT system, using human GAPDH as an endogenous control. The ΔΔ Ct method was used for calculating expression levels.

### Proliferation assay

Cell proliferation was determined using CyQUANT Cell Proliferation Assay (Invitrogen) according to the manufacturer's protocol. The fluorescence intensity was measured using a fluorescence microplate reader (Molecular Devices, Sunnyvale, CA).

### Migration assay

The migratory ability of thyroid cancer cells was assessed by the scratch wound assay in cells grown in monolayer. Approximately 150,000 cells were transfected with pre-miR-886-3p (25 nM) or miR-NC (25 nM) then plated in 6-well plates and allowed to attach and grow for 44 hours. Thereafter, three vertical wounds were made with a sterile 10 µl pipette tip and a horizontal line was made across the three lines to allow observation of cells at the same point. The cells were inspected every 6 hours and measurements taken up to 22 hours from the initial wound.

### Genome-wide mRNA expression microarray

TPC-1 cells were transfected with pre-miR-886-3p and pre-miR-NC. Seventy-two hours after transfection, cells were washed three times with PBS. Total RNA was prepared from triplicate cell cultures using the RNeasy (Qiagen, Valencia, CA) kit according to the manufacturer's instruction. RNA quality was ensured, before labeling, using the Agilent RNA 6000 Nano kit and the Bioanalyzer 2100. One hundred fifty ng of total RNA was used to perform cDNA reverse transcription, synthesis, amplification, fragmentation and terminal labeling with the GeneChip WT Sense Target Labeling and Control Reagents (Affymetrix, Santa Clara, CA). Approximately 25 ng/µL of cDNA was hybridized to the Affymetrix Human Gene 1.0 ST Array GeneChip. The arrays were washed and stained using the fluidics protocol FS450_0007 procedure on an Affymetrix Fluidics Station 450. The probe intensities were scanned by the GeneChip Scanner 3000. The raw data was normalized and analyzed using Partek Genomic Suite (Partek Inc., St Louis, MO, USA). Analysis of variance was used to determine those probe sets significantly different between the two groups. The gene list was filtered with a fold-change cutoff of 2. This resulted in output of gene list with genes that have significant differential expression at P≤0.001 and 2-fold or more differences. Pathway analysis was performed using the DAVID bioinformatics resources.

### Cell cycle flow cytometry analysis

The effects of miR-886-3p overexpression on cell cycle progression were assessed using propidium iodide flow cytometry. Briefly, TPC-1 and FTC-133 cells were transfected with pre-miR-886-3p or pre-miR-NC for 72 hours to 120 hours. The cells were washed with PBS, harvested, and fixed in 70% ethanol. Then the cells were treated with (500 U/mL) DNase-free RNase and stained with propidium iodide. Cell samples were analyzed on a FACSCalibur (BD Biosciences, San Jose, CA), and G_0_G_1_, S, and G_2_M phase fractions were determined using ModFitLT software (Topsham, ME).

### Apoptosis flow cytometry analysis

The effects of miR-886-3p overexpression on cell death were assessed by Annexin V- FITC and propidium iodide flow cytometry using ApoAlert Annexin V kit (Clontech, Mountain View, CA). TPC-1 and FTC-133 cells were transfected with pre-miR-886-3p or pre-miR-NC for 72 hours to 120 hours. The cells were harvested and stained with Annexin V- FITC and propidium iodide according to the manufacturer's protocol. Cell samples were analyzed on a FACSCalibur, and apoptotic fractions were determined.

### Luciferase Reporter Assay

The 1160 base pair 3′-UTR of *CDC6* was cloned into the empty luciferase reporter vector pEZX-MT01 (GeneCopoeia, Rockville, MD), generating a wild-type *CDC6* UTR luciferase reporter construct (pEZX-WT-UTR). Mutations in the 3′-UTR of *CDC6* were designed for the first four nucleotides (CACC to GTGG) or the last three nucleotides (CGC to GCG) of the 7-mer seed region of the putative binding site of miR-886-3p, generating two mutants termed as pEZX-Mut-UTR-01 and pEZX-Mut-UTR-02, respectively. All constructs were sequence-verified by DNA sequencing. For the dual luciferase assay, TPC-1 cells were plated in triplicate into 12-well plates and co-transfected with 0.25 µg of the reporter construct and 15 pmol of pre-miR-886-3p or pre-miR-NC by using Lipofectamine 2000 (Invitrogen). At 24 hours, the cells were lysed and assayed for both firefly and renilla luciferase using Luc-Pair™ miR Luciferase Assay Kit (GeneCopoeia, Rockville, MD) on a SpectraMax M5e microplate reader (Molecular Device, Sunnyvale, CA) according to the manufacturers' instructions.

### Data Analysis

Data is presented as the mean ± standard error of the mean. To determine statistical significance, the Mann-Whitney U test, t test and analysis of variance were used, as appropriate. A p-value of less than 0.05 was considered statistically significant. All experiments were repeated at least three times.

## Results

### MiRNA profiling of sporadic and familial papillary thyroid cancer

**Figure 1:**
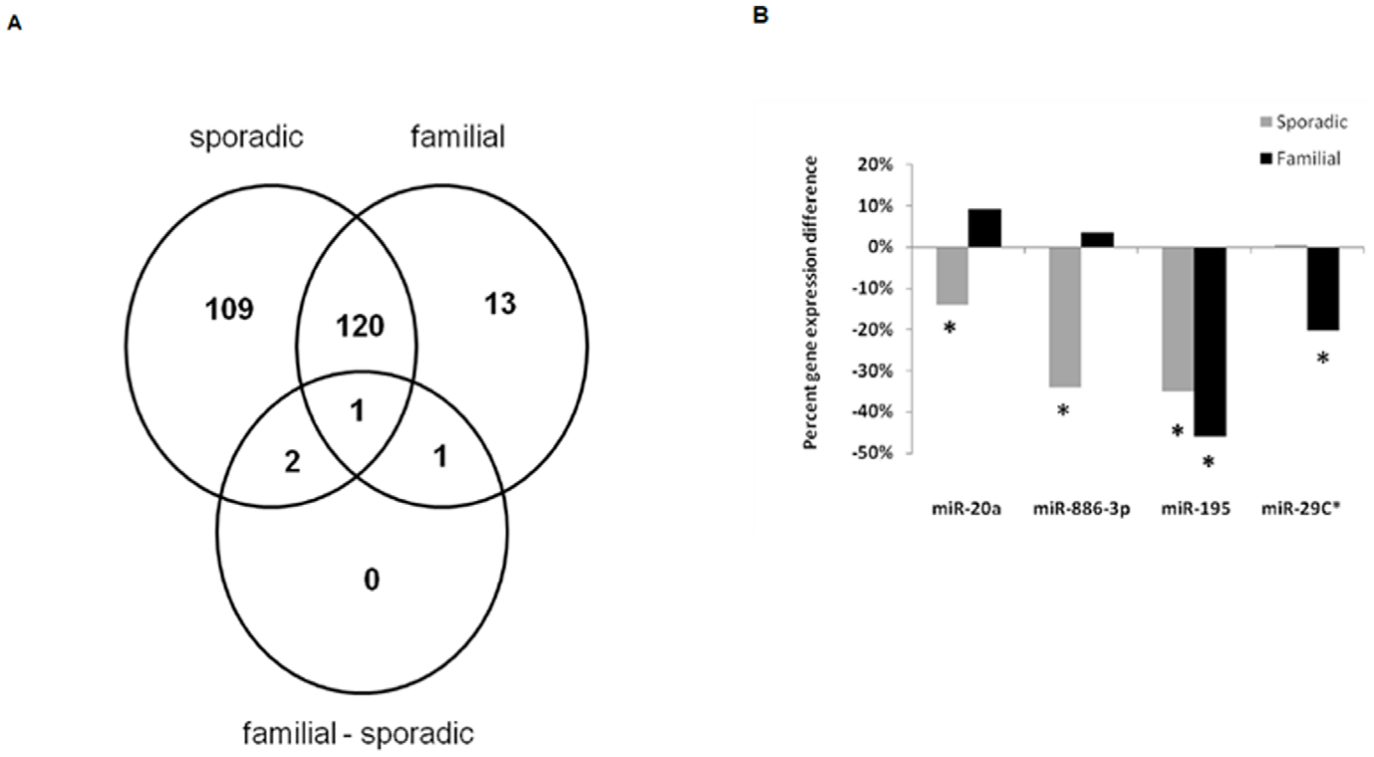
MiRNA expression in thyroid tissue. **A**) Comparison of differentially expressed miRNAs in sporadic and familial papillary thyroid cancer compared to normal thyroid tissue. The sporadic circle indicates this group's comparison to normal thyroid tissue, the familial circle indicates this group's comparison to normal thyroid tissue, and the familial-sporadic circle indicates comparison between familial and sporadic tumors. **B**) Relative percent miRNA expression difference between familial and sporadic tumors normalized to normal tissue samples. * p value<0.05.

**Figure 2:**
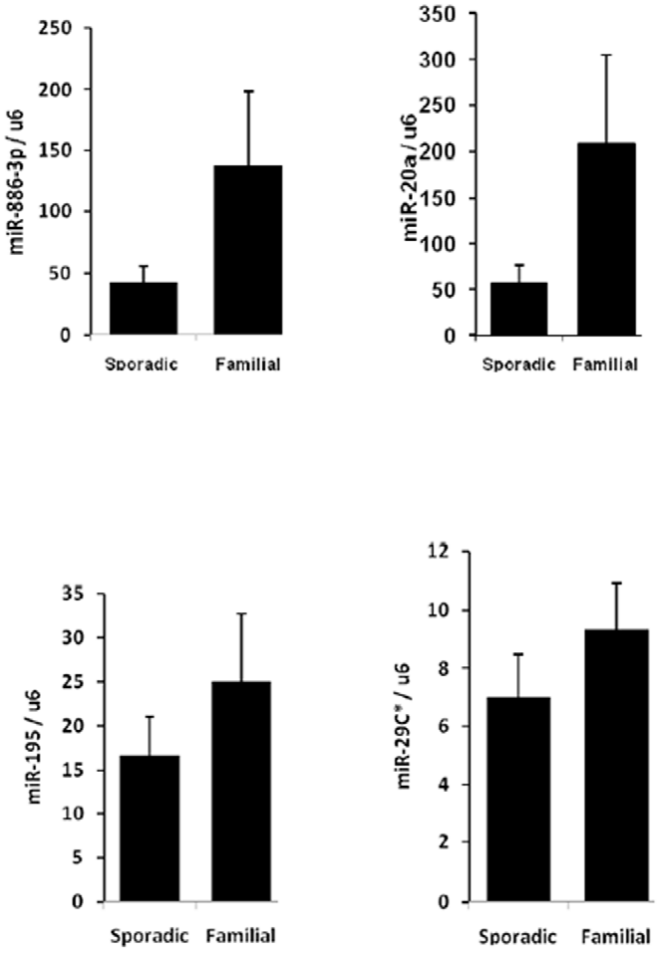
Validation of dysregulated miRNAs in sporadic versus familial papillary thyroid cancer by quantitative RT-PCR. MiR-20a was 4-fold and miR-886-3p was 3-fold higher in familial papillary thyroid cancer as compared to sporadic (p = 0.025 for miR-20a, p = 0.028 for miR-886-3p, p = 0.37 for miR-195, p = 0.46 for miR-29c). Values are average expression plus and minus the standard error of mean. The Y axis represents the ratio of miRNA and U6 RNA using 2^−ΔΔCt^ method, and the lowest value of the sample was set to 1.0.

We compared the miRNA expression profile of familial and sporadic papillary thyroid cancer tumor samples using whole-genome miRNA microarray analysis. The samples were matched based on the age and gender of the patients, and the TNM tumor stage. MiRNA expression in tumor samples was compared to normal thyroid tissue samples. There were 232 miRNAs in sporadic and 135 miRNAs in familial papillary thyroid cancer which were dysregulated compared to normal thyroid tissue samples (**Figure 1A**) (p<0.05). One hundred nine of the dysregulated miRNAs were unique to sporadic, 13 were unique to familial, and four miRNAs were significantly differentially expressed between sporadic and familial papillary thyroid cancer (**Figure 1A and 1B**). Two of the four miRNAs were validated to be significantly differentially expressed between familial and sporadic papillary thyroid cancer by quantitative real time RT-PCR (**Figure 2**). Both miR-20a and miR-886-3p were also significantly downregulated in sporadic papillary thyroid cancer compared to normal thyroid tissue (p = 0.032 and p = 0.0032, respectively) (**Figure 1B**).

### Target genes and pathways of MiR-886-3p in thyroid cancer cells

**Figure 3:**
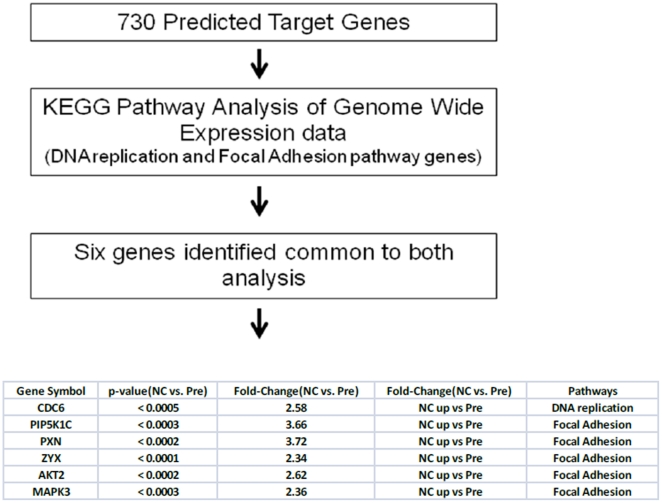
Work flow of integrated genomic analysis of miR-886-3p target genes and pathways.

Given the function of miR-886-3p in tumor cell biology is unknown, we were first interested in determining the target gene(s) and pathway(s) that may be regulated by miR-886-3p. We used two approaches to determine miR-886-3p targets: miRNA target prediction and genome-wide mRNA expression analysis in cells overexpressing miR-886-3p (**Figure 3**). We found 730 predicted target genes for miR-866-3p using target prediction databases (miRanda, miRBase, Target ScanS). By performing KEGG pathway analysis on the mRNAs dysregulated in miR-886-3p less under expressed cells, we found genes involved in the DNA replication and focal adhesion pathways were misexpressed upon miR-886-3p overexpression in thyroid cancer cell lines. Integration of these bioinformatics approaches identified six genes in common between the analyses. These genes are therefore predicted to be miR-886-3p targets.

**Figure 4:**
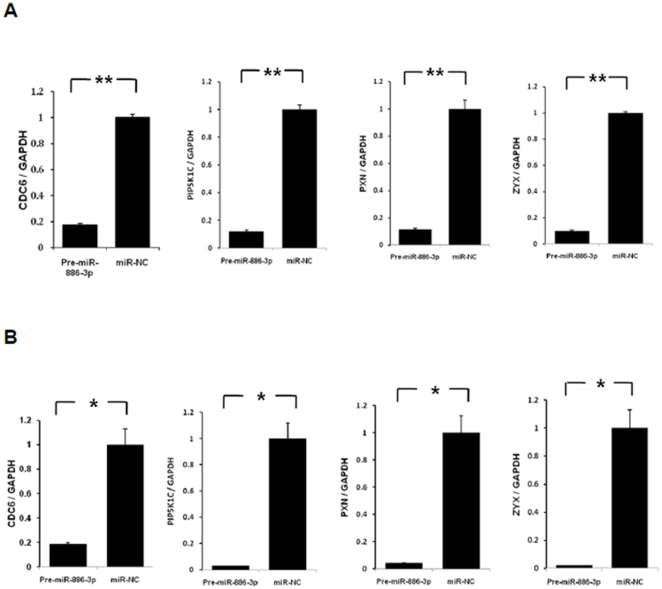
Validation of 4 target genes of miR-886-3p by quantitative RT-PCR. Transfection of pre-miR-886-3p significantly decreased the mRNA levels of four target genes (*CDC6*, *PIP5K1C*, *PXN*, *ZYX*) in **A**) TPC-1 cell line and **B**) FTC-133 cell line (*p<0.05; ** p<0.01). The Y axis represents the ratio of the specific target gene and GAPDH using 2^-ΔΔCt^ method, and the value of the miR-NC group was set at 1.0 to allow comparison of fold differences among different cell lines and genes.

**Figure 5:**
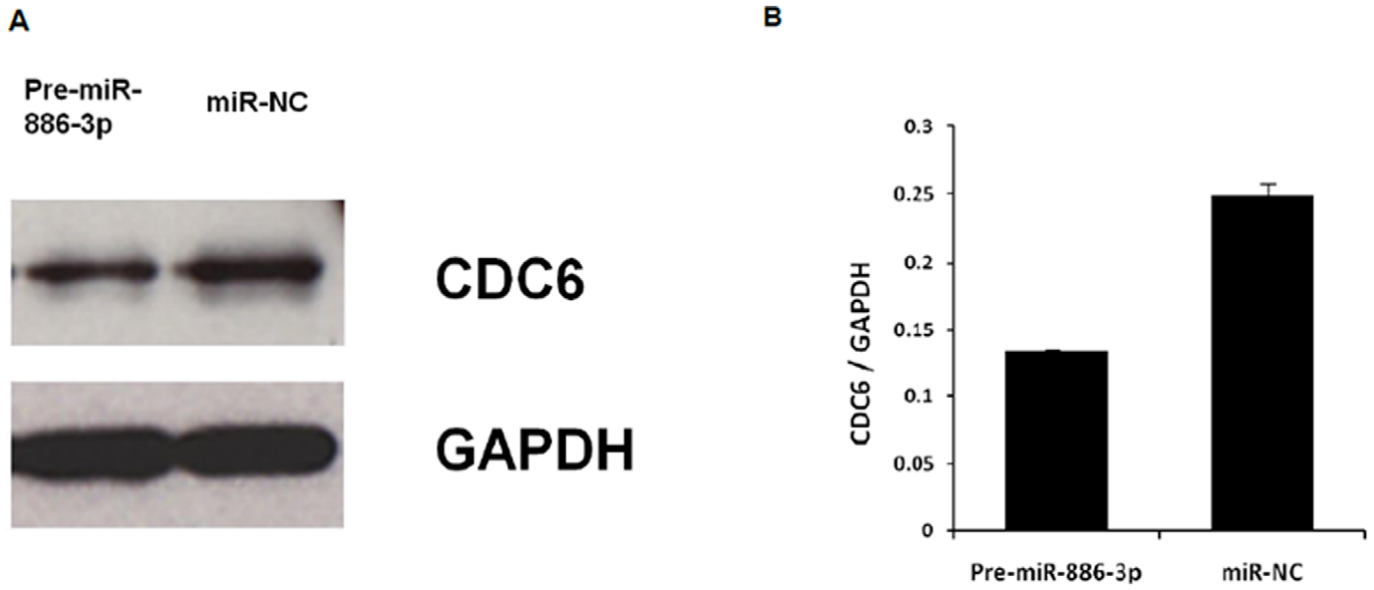
Western blot analysis of *CDC6* protein expression with pre-miR-886-3p overexpression in TPC-1 cells. **A**) Representative western blot image of *CDC6* protein expression at 72 hours after transfection with pre-miR-886-3p as compared to negative control (miR-NC). **B**) Band densitometry quantification of *CDC6* protein expression. The software ImageJ (Maryland, USA) was used for densitometric analysis of western blots.

**Figure 6:**
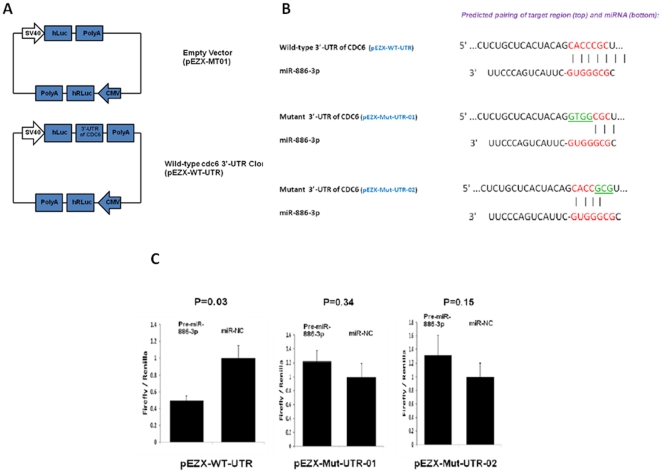
Analysis of miR-886-3p effect on 3′-UTR of *CDC6*. **A**) pEZX-WT-UTR vector with both Renilla luciferase (hRLuc) was used as an internal control and firefly luciferase (hLuc) was upstream of the 3′-UTR construct. **B**) Putative binding site of miR-886-3p on the *CDC6* 3′ UTR along with the mutations in the predicted seed region. **C**) Left figure shows luciferase activity of pEZX-WT-UTR in TPC-1 cells when co-transfected with pre-miR-886-3p or pre-miR-NC, p<0.05. Middle and right figures showluciferase activity of pEZX-Mut-UTR-01 and pEZX-Mut-UTR-02 in TPC-1 cells when co-transfected with pre-miR-886-3p or pre-miR-NC, respectively. All luciferase measurements were made in triplicates and readings were performed at 24 hours post-transfection.

To further address this hypothesis, we selected four of these genes for validation upon miR-886-3p overexpression. We found significant downregulation of all four genes upon miR-886-3p overexpression by quantitative RT-PCR (**Figure 4**). Of these genes, we were particularly interested in *CDC6* since this gene encodes a potent regulator of DNA replication and oncogenesis [27]. Interestingly, western blot analysis showed downregulation of *CDC6* protein expression within 72 hours of miR-886-3p overexpression (**Figure 5**). To further assess if *CDC6* is a direct target of miR-886-3p regulation, transfection of the 3′UTR *CDC6* wild type vector in thyroid cancer cells with miR-886-3p overexpression showed significant downregulation of luciferase activity suggesting that *CDC6* was a direct target of miR-886-3p (**Figure 6**). Mutations in the predicted seed region for miR-886-3p in the 3′UTR of *CDC6* abolished this effect, further suggesting that miR-886-3p directly regulates *CDC6* expression (**Figure 6**).

### MiR-886-3p regulates cell cycle and proliferation, and spheroid formation and migration

**Figure 7:**
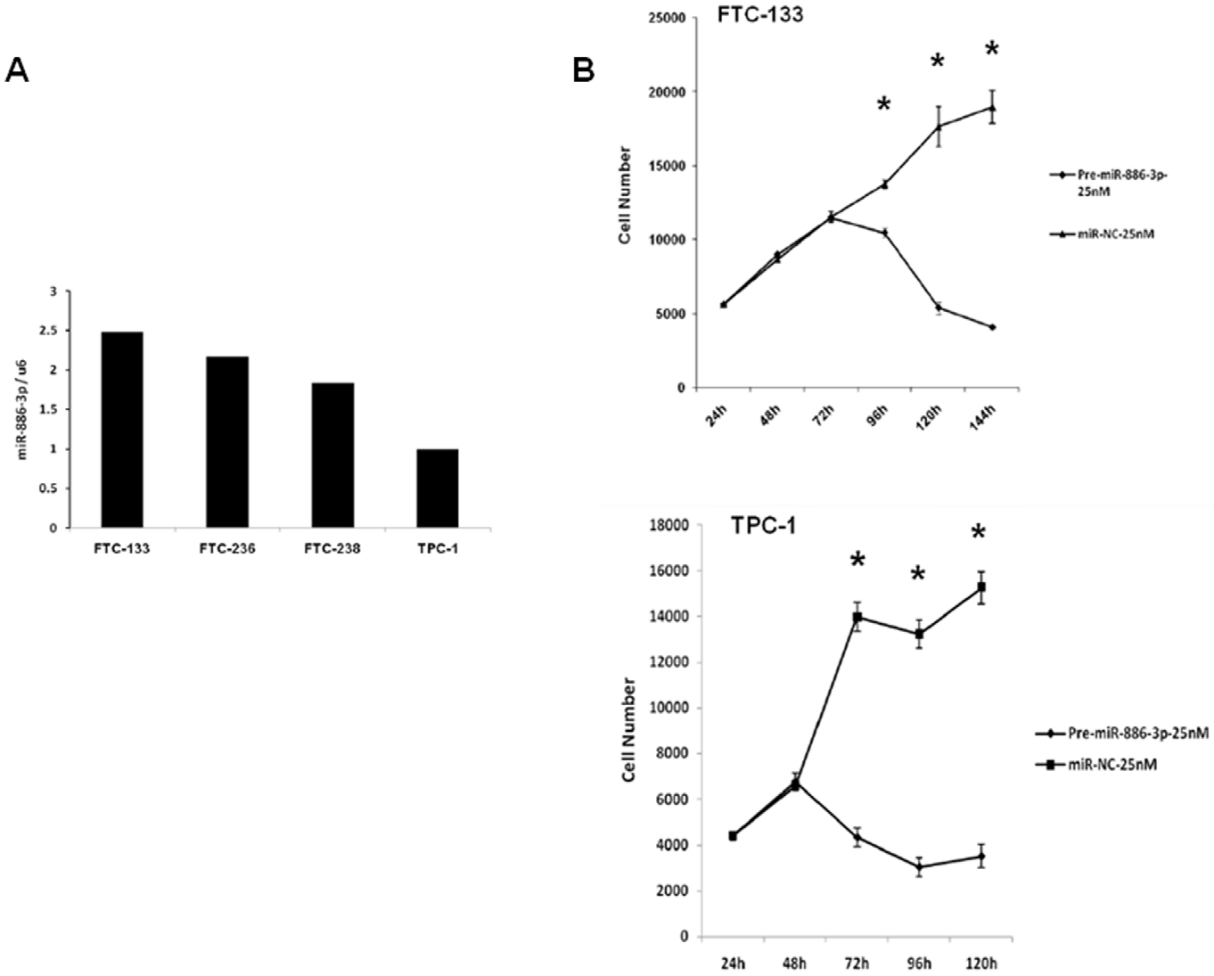
MiR-886-3p expression levels and function in thyroid cancer cell lines. **A**) Baseline expression of miR-886-3p in four thyroid cancer lines. **B**) Effect of miR-886-3p overexpression on thyroid cancer cell proliferation. Pre-miR-886-3p significantly inhibited thyroid cancer cell proliferation. Error bars represent standard error of mean. *p≤0.001.

Because we found miR-886-3p regulates genes involved in DNA replication and focal adhesion pathways, we were interested in determining the role of miR-886-3p on cell cycle and cellular proliferation, as well as, spheroid formation and migration. We first determined the basal expression of miR-886-3p in four well-characterized and authenticated thyroid cancer cell lines and found that FTC-133 and TPC-1 had highest and lowest level of miR-886-3p expression, respectively (**Figure 7A**). We used these cells lines to analyze the effect of miR-886-3p on cell proliferation. Overexpression of miR-886-3p significantly inhibited cell proliferation by 79% in FTC-133 cells at 144 hours (p<0.001) and 77% in TPC-1 cells at 120 hours (p<0.001) as compared to pre-miR-NC (**Figure 7B**).

Given the profound effect of miR-886-3p overexpression on cellular proliferation, we wanted to next explore the mechanism of miR-886-3p-mediated growth inhibition. We, thus, performed flow cytometry and Annexin V assays to determine the effect of miR-886-3p on cell cycle progression and apoptosis, respectively. Overexpression of miR-886-3p significantly increased the number of cells in S phase (12–16%) while decreasing the number of cells in G_0/_G_1_ (11–22%) (p<0.001). We, however, found no significant increase in the number of cells in apoptosis with and without miR-886-3p overexpression. This data indicates that miR-886-3p regulates cell cycle and proliferation consistent with our miR-886-3p pathway and target prediction analyses.

**Figure 8:**
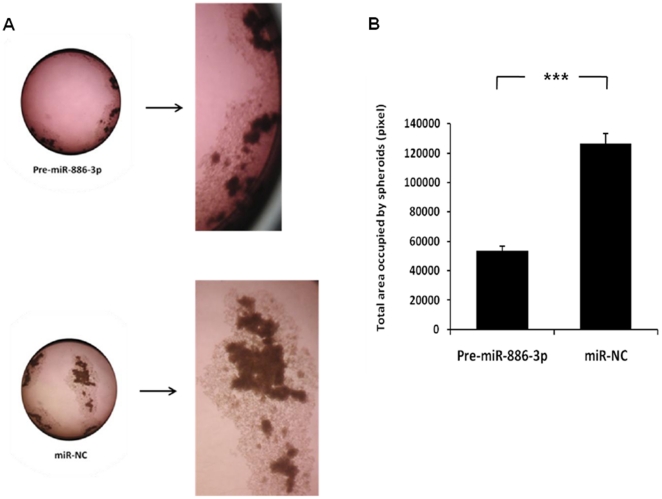
Effect of miR-886-3p overexpression on thyroid cancer cell spheroid formation. Pre-miR-886-3p decreased the size and number of spheroids in the FTC-133 cell line. **A**) Representative image of spheroids in culture with miR-886-3p overexpression. **B**) Quantification of spheroid difference with miR-886-3p overexpression. The total area occupied by spheroids within an image was measured by circumscribing the perimeter of each spheroid, marking the entire area, and calculating the pixel numbers by using ImageJ software (Maryland, USA). The experiments were repeated three times, and similar results were observed. (*** indicates p<0.001).

**Figure 9:**
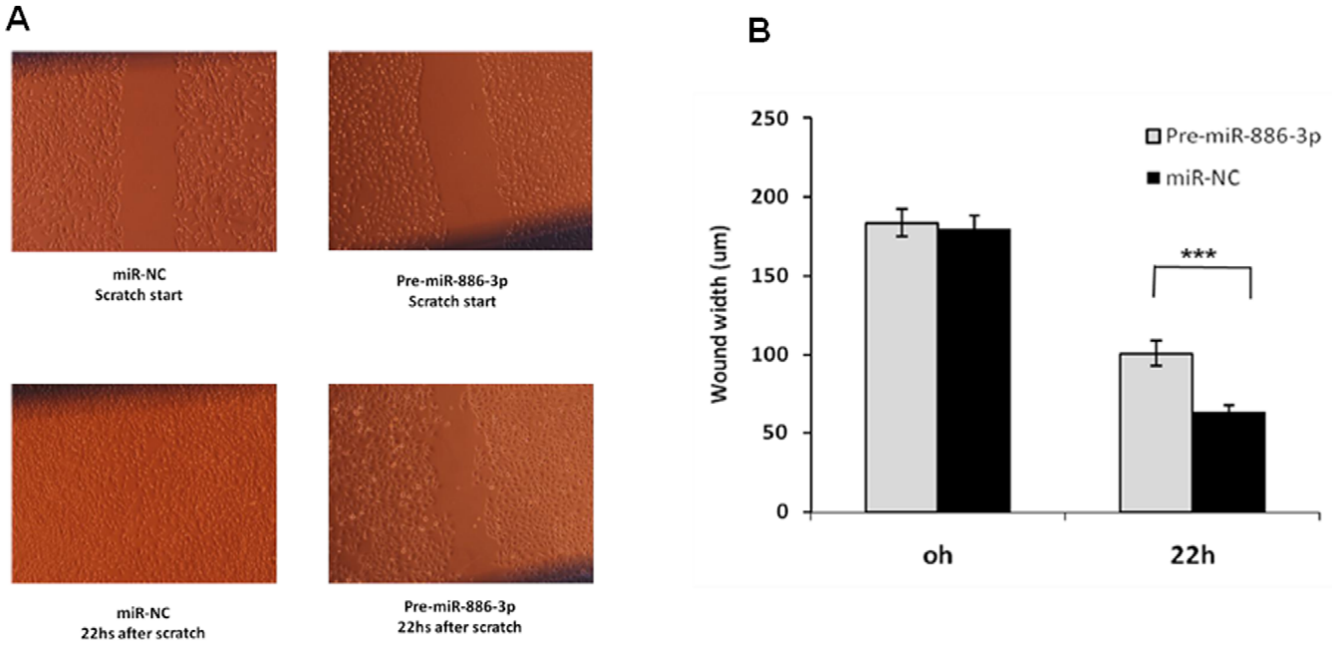
Overexpression of miR-886-3p inhibited migration of TPC-1 cells. Pre-miR-886-3p significantly decreased cell migration at 22 hours (p<0.001). **A**) Representative image of wound assay at time 0 and 22 hours after scratch. **B**) Quantification of wound width closure with miR-886-3p overexpression. Six different locations were visualized and photographed under a phase-contrast inverted microscope (10× magnification) in each plate at different time points, and three plates were used for each group. The wound width in the 10× images was measured using a standard caliper. The experiments were repeated three times, and similar results were observed. (*** indicates p<0.001).

Because miR-886-3p pathway and target analyses showed genes regulating focal adhesion were misexpressed, we also determined the effect of miR-886-3p overexpression on thyroid cancer cell line spheroid formation and cellular migration. The FTC-133 cell line forms spheroids when cultured in ultralow adherent culture flasks within 72 hours. Consistent with the effect of miR-886-3p overexpression in monolayer cells, we observed a decrease in the number and size of spheroids in the FTC-133 thyroid cancer cell line which was sustained for up to 2 weeks in culture (**Figure 8**). Additionally, miR-886-3p overexpression decreased the multiple cellular branch-like structures observed in the negative control cells. We next determined the effect of miR-886-3p overexpression on cellular migration using the scratch wound assay. Overexpression of miR-886-3p significantly inhibited migration of the TPC-1 cell line (p<0.001) (**Figure 9**).

## Discussion

In this study, we performed miRNA profiling of familial and sporadic papillary thyroid cancer tumor samples. We found four miRNAs are differentially expressed between these groups, two of which, miR-886-3p and miR-20a, were validated to be differentially expressed by 3- and 4-fold, respectively. Both of these miRNAs were downregulated in papillary thyroid cancer as compared to normal thyroid tissue by 3.5–4-fold. Genome-wide gene expression pathway and target prediction analyses demonstrated genes involved in DNA replication and focal adhesion pathways were targeted by miR-886-3p. Overexpression of miR-886-3p in thyroid cancer cell lines significantly inhibited cellular proliferation, the number and size of spheroids, and cellular migration. Moreover, overexpression of miR-886-3p increased the number of cells in S phase and decreased the number of cells in G_0_G_1_ phase with no effect on apoptosis.

We are not aware of any other studies which have compared the miRNA profile of tumors associated with familial cancer syndromes with their sporadically occurring counterparts. Such an analysis provides important insights into the genetic pathways that may be different in histologically similar tumors, the important targets of predisposing genetic changes, and, in the case of FNMTC, the molecular basis. Although most investigators have suggested that FNMTC is a distinct hereditary syndrome, arguments against this include that, 1) the same susceptibility gene(s) and or loci have not been observed across several linkage studies, 2) nonmalignant thyroid tumors are common and thus a thyroid cancer diagnosis may be a founder effect or incidentally discovered, and 3) the expressivity is variable [2]. On the other hand, several studies support FNMTC as being a distinct hereditary syndrome because, 1) multiple kindreds have been reported to have multigenerational occurrence of FNMTC, 2) there is a higher rate of thyroid cancer in men and children within FNMTC pedigrees, 3) there is an earlier age of presentation, and 4) there is male-to-male transmission. Lastly, epidemiologic studies demonstrate that the risk of thyroid cancer in a first-degree relative of a patient diagnosed with thyroid cancer is 5-fold higher [28]. Given this controversy, we set out to determine if the expression profiles of familial and sporadic papillary thyroid cancer were different. A large number of miRNAs were dysregulated in both familial and sporadic papillary thyroid cancer compared to normal thyroid tissue. We performed the miRNA profiling in tumor samples from patients which were matched for age, gender and TNM cancer stage. Such an approach was used to minimize confounding factors which could result in different miRNA expression profile due to different extent of disease, histologic subtypes of tumor, and differentiation status of tumor [29], [30], [31], [32], [33], [34], [35]. Therefore, we believe our careful study design and analysis provide findings which, for the first time, support a molecular difference in familial and sporadic papillary thyroid cancer.

Among the differentially expressed miRNAs between familial and sporadic papillary thyroid cancer, two miRNAs were validated to be differentially expressed by quantitative RT-PCR. Both miR-20a (13q31.3) and miR-886-3p (5q31.2) are not located in chromosomal loci previously identified as susceptibility loci by linkage studies in kindreds with FNMTC. Given the small nucleotide lengths of miRNAs, it is not surprising that genetic alterations in the chromosomal loci encoding miR-20a and miR-886-3p may have been missed in these studies, even with the use of high resolution SNP arrays [13]. In the future, germ line sequence analysis can be used to determine if these miRNAs are candidate susceptibility genes for FNMTC. Additionally, it will be important to determine in future studies the mechanism of the general MiR-886-3p downregulation in thyroid cancer is due to copy number variation, inactivating mutations or deletion.

We were interested in studying the role of miR-886-3p in thyroid carcinogenesis for several reasons. Mainly, the function of miR-886-3p is unknown, the miR-886 gene's chromosomal location on 5q31 is shared with transforming growth factor β1, an important regulator in epithelial carcinogenesis, and the level of miR-886-3p expression difference between familial and sporadic papillary thyroid cancer was large (3-fold). Consistent with our findings, a recent study showed miR-886-3p was downregulated in squamous cell lung cancer compared with normal lung tissue, suggesting a potential tumor suppressor role for this miRNA [5]. Using two well-characterized thyroid cancer cell lines with differing levels of basal miR-866-3p expression, we demonstrated that miR-886-3p plays a critical role in cellular proliferation and migration, and regulates genes involved in the DNA replication and focal adhesion pathways. We selected *CDC6*, a potent regulator of DNA replication and oncogenesis [27], for further analysis and found that *CDC6* was a direct target of miR-886-3p. Although our functional studies were done in thyroid cancer lines, our data suggests that miR-886-3p is an important regulator of tumor cell biology in thyroid cancer [26].

Overexpression of miR-886-3p caused dramatic changes in expression of genes which regulate DNA replication and focal adhesion. Four genes (*CDC6*, *PIP5K1C*, *PXN*, *ZYX*) had >4-fold knockdown with increased miR-886-3p expression. *CDC6* encodes a protein essential for DNA replication initiation and mitosis, and thus likely accounts for the increased number of cells in S phase after overexpression of miR-886-3p [36]. *PIP5K1C* and PXN are necessary for cellular adhesion in a variety of cell types [37], [38]. ZYX is a phosphoprotein which is concentrated at focal adhesions and along the actin cytoskeleton and regulates cellular adhesion and migration [39], [40], [41], [42], [43], [44]. The effect of these and other genes in the two pathways identified which are targeted by miR-886-3p, are consistent with the dramatic effect on cellular proliferation and migration observed in our miR-886-3p functional studies.

MiR-20a expression has been characterized in several human malignancies and is dysregulated in prostate, ovarian, head and neck squamous cell, and colon cancers [34], [45], [46], [47]. In epithelial cells, miR-20a promotes cellular proliferation and invasion, and higher expression levels are associated with tumor dedifferentiation [34], [48]. Although previous studies of miRNA profiling in thyroid tumors have not identified miR-20a to be one of the most dysregulated miRNAs, in our comparison of familial and sporadic papillary thyroid cancer, it was significantly downregulated in thyroid cancer [49]. Taken together with its known function as a growth promoting miRNA and the effects of miR-886-3p, our findings suggest that these two miRNAs may play a role in the different clinical feature and tumor biology observed in familial versus sporadic cases of papillary thyroid cancer [2], [6], [15].

In summary, our findings suggest that miR-886-3p plays an important role in thyroid cancer tumor cell biology and may account for the clinical manifestation of non-medullary thyroid cancer in sporadic versus familial cases. To our knowledge, this is the first study to identify a role for miR-886-3p in regulating tumor cell biology, specifically in thyroid cancer cells.
